# Supplementary material for: Circulating Omega-3 Polyunsaturated Fatty Acids Levels in Coronary Heart Disease: Pooled Analysis of 36 Observational Studies
Source: Nutrients. 2024 May 24;16(11):1610. doi: 10.3390/nu16111610 (PMC11174367; doi:10.3390/nu16111610)
Supplement: Supplementary file 1 [file nutrients-16-01610-s001.zip › nutrients-2990085-supplementary.pdf]

Supplementary Materials:
Omega-3 polyunsaturated fatty acids levels in coronary heart disease: an individual-level pooled analysis of 36 observational studies

Yanan Xiao, Yifang Chen, Anne Pietzner, Ulf Elbelt, Zhimin Fan, Karsten H. Weylandt

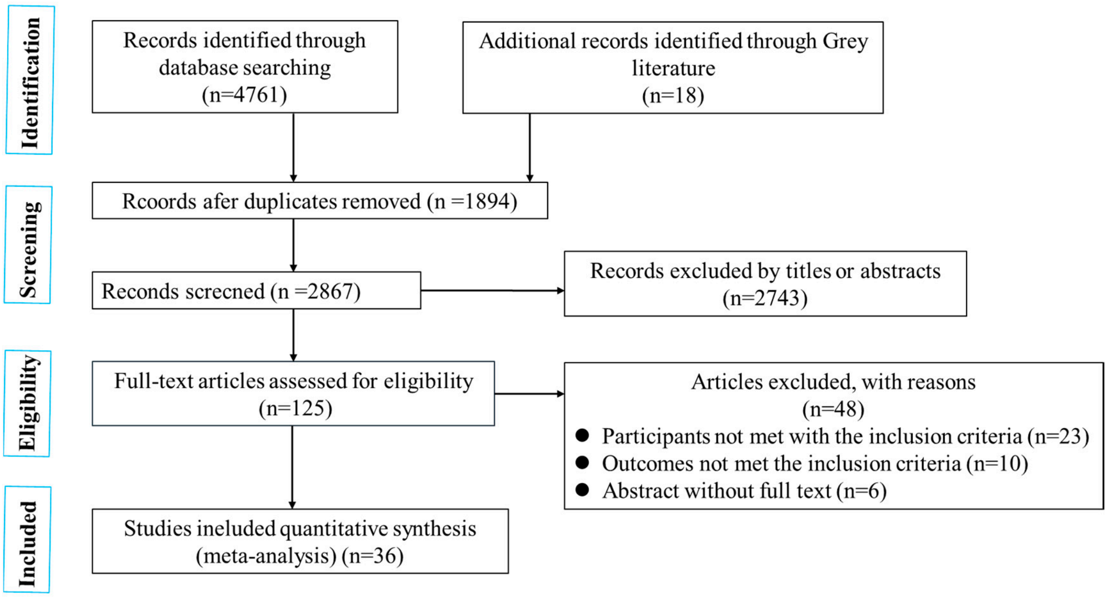

Figure S1 Flow chart of included studies

Table S1. Search strategy

**PubMed**	(Omega-3 Fatty Acid OR Omega 3 Fatty Acid OR Omega-3 Fatty Acids OR n-3 Oil OR n-3 OR n 3 Oil OR n3 Oil OR n3 OR n-3 Fatty Acids OR n 3 Fatty Acids OR Omega 3 Fatty Acids OR n-3 PUFA OR n 3 PUFA OR n3 Fatty Acid OR n3 OR n3 PUFA OR n3 Polyunsaturated Fatty Acid OR n3 Oils OR n-3 Oils OR n 3 Oils OR N-3 Fatty Acid OR N-3 Fatty OR Fatty Acid OR N 3 Fatty Acid OR n-3 Polyunsaturated Fatty Acid OR n 3 Polyunsaturated Fatty Acid OR Eicosapentaenoic Acid OR EPA OR Docosahexaenoic Acid OR DHA OR Fish oil)<br>AND<br>(Coronary heart disease OR Cardiovascular Diseases OR Vascular Diseases OR Myocardial Ischemia OR Coronary Disease OR Coronary Artery Disease OR Coronary Occlusion OR Coronary Thrombosis OR Coronary Vasospasm OR Heart Diseases OR Coronary Stenosis OR Acute coronary syndrome)<br>AND<br>(Prospective study [pt] OR Case-Control Study [pt] OR Epidemiologic Study [mh] OR Cohort Study [mh] OR Nested case-control study [mh] OR Case-Comparison Study[mh] OR Matched Case-Control Study)
**EMBASE/Cochrane Central Register of Controlled Trials (CENTRAL)/Web of Science**	#1: Omega-3 Fatty Acid OR Omega 3 Fatty Acid OR Omega-3 Fatty Acids OR n-3 Oil OR n-3 OR n 3 Oil OR n3 Oil OR n3 OR n-3 Fatty Acids OR n 3 Fatty Acids OR Omega 3 Fatty Acids OR n-3 PUFA OR n 3 PUFA OR n3 Fatty Acid OR n3 OR n3 PUFA OR n3 Polyunsaturated Fatty Acid OR n3 Oils OR n-3 Oils OR n 3 Oils OR N-3 Fatty Acid OR N-3 Fatty OR Fatty Acid OR N 3 Fatty Acid OR n-3 Polyunsaturated Fatty Acid OR n 3 Polyunsaturated Fatty Acid OR Eicosapentaenoic Acid OR EPA OR Docosahexaenoic Acid OR DHA OR Fish oil<br>#2: fatty acid*<br>#3: Coronary heart disease OR Cardiovascular Diseases OR Vascular Diseases OR Myocardial Ischemia OR Coronary Disease OR Coronary Artery Disease OR Coronary Occlusion OR Coronary Thrombosis OR Coronary Vasospasm OR Heart Diseases OR Coronary Stenosis OR Acute coronary syndrome)<br>#4: Coronary* OR Cardiovascular* OR Heart*<br>#5: Prospective*.tw. OR Case-Control* OR Epidemiologic Study [mh] OR Cohort Study [mh] OR Nested case-control study [mh] OR Case-Comparison Study[mh] OR Matched Case-Control Study<br>#6: #1 OR #2<br>#7: #3 OR #4<br>#8: #5 AND #6 AND #7

Table S2. Newcastle-Ottawa Scale (NOS) for quality assessment of the studies

Study	Selection				Comparability		Outcome			Total
	1	2	3	4	5a	5b	6	7	8	
**Cohort**										
Sun et.al (2016)	*	*	*	*	*	*	*	*	*	9
Lemaitre et.al (2003)	*	*	*	*		*	*	*	*	8
Matthan et.al (2014)	*	*	*	*	*	*	*	*	*	9
Simon et.al (1995)			*	*	*	*	*	*	*	7
Khaw et.al (2012)	*	*	*	*	*	*	*	*	*	9
Papandreou et.al (2019)	*	*	*	*	*	*	*	*	*	9
Erkkilä et.al (2003)	*	*	*	*	*	*	*	*	*	9
Zelniker et.al (2021)	*	*	*	*	*	*	*	*		8
Chei et.al (2018)	*	*	*	*	*	*	*	*	*	9
Otto et.al (2013)	*	*	*	*	*	*	*	*	*	9
Goede et.al (2013)	*	*	*	*	*	*	*	*	*	9
Guallar et.al (1995)		*	*	*		*	*	*	*	7
Sun et.al (2008)	*	*	*	*	*	*	*	*	*	9
Mozaffarian et.al (2013)	*	*	*	*	*	*	*	*		8
Harris et.al (2018)	*	*	*	*	*	*	*	*	*	9
Harris et.al (2007)	*		*	*	*	*	*	*		8
Block et.al (2008)	*	*	*	*	*	*	*	*	*	8
Albert et.al (2002)	*	*	*	*		*	*	*	*	8
Hamazaki et.al (2018)	*	*	*	*	*	*	*	*	*	8
Liu et.al (2019)	*	*	*	*	*	*	*	*		8
**Case-control**										
Lea et.al (1982)		*	*	*		*	*	*		6
Luostarinen et.al (1993)		*	*	*		*	*			5
Prisco et.al (1986)	*	*	*	*			*	*		6
Roberts et.al (1993)		*	*	*	*	*	*			6
Oda et.al (2005)	*	*	*	*	*		*	*	*	8
Jama et.al (2002)	*	*	*	*	*		*		*	7
Marangoni et.al (2014)		*	*	*	*	*	*	*	*	8
Rhee et.al (2008)		*	*	*	*	*	*		*	7
Lemaitre et.al (2009)	*	*	*	*	*		*	*	*	8
Freije et.al (2009)	*	*	*	*			*	*	*	7
Ahmed et.al (2017)	*	*	*	*	*	*	*	*	*	9
Pedersen et.al (2000)	*	*	*	*	*		*	*	*	8
Skuladottir et.al (1988)		*	*	*		*	*		*	6
Kirkeby et.al (1972)		*	*	*		*	*			5
Siscovick et.al (1995)	*	*	*	*	*		*			6
Lopes et.al (2007)	*	*	*	*	*		*	*		7

Selection: 1) representativeness of the exposed study; 2) selection of the non-exposed study; 3) ascertainment of exposure; 4) demonstration that outcome of interest was not present at start of study ; Comparability: comparability of studies on the basis of the design or analysis controlled for confounders: for the most important factor (5a),for other factors (5b); Outcome: 6) assessment of outcome; 7) sufficient follow-up time; 8) adequacy of follow-up.

Cohort:

1. Sun, Y., et al., *Plasma α-linolenic and long-chain ω-3 fatty acids are associated with a lower risk of acute myocardial infarction in Singapore Chinese adults.* The Journal of Nutrition, 2016. **146**(2): p. 275-282.
2. Lemaitre, R.N., et al., *n− 3 Polyunsaturated fatty acids, fatal ischemic heart disease, and nonfatal myocardial infarction in older adults: the Cardiovascular Health Study.* The American journal of clinical nutrition, 2003. **77**(2): p. 319-325.
3. Matthan, N.R., et al., *Plasma phospholipid fatty acid biomarkers of dietary fat quality and endogenous metabolism predict coronary heart disease risk: a nested case-control study within the Women's Health Initiative Observational Study.* Journal of the American Heart Association, 2014. **3**(4): p. e000764.
4. Simon, J.A., et al., *Serum fatty acids and the risk of coronary heart disease.* American journal of epidemiology, 1995. **142**(5): p. 469-476.
5. Khaw, K.-T., et al., *Plasma phospholipid fatty acid concentration and incident coronary heart disease in men and women: the EPIC-Norfolk prospective study.* PLoS medicine, 2012. **9**(7): p. e1001255.
6. Papandreou, C., et al., *Association Between Fatty Acids of Blood Cell Membranes and Incidence of Coronary Heart Disease: A Case-Control Study Nested in the PREDIMED Trial.* Arteriosclerosis, Thrombosis, and Vascular Biology, 2019. **39**(4): p. 819-825.
7. Erkkilä, A.T., et al., *n− 3 fatty acids and 5-y risks of death and cardiovascular disease events in patients with coronary artery disease.* The American journal of clinical nutrition, 2003. **78**(1): p. 65-71.
8. Zelniker, T.A., et al., *Plasma omega-3 fatty acids and the risk of cardiovascular events in patients after an acute coronary syndrome in MERLIN-TIMI 36.* Journal of the American Heart Association, 2021. **10**(8): p. e017401.
9. Chei, C.-L., et al., *Serum fatty acid and risk of coronary artery disease—Circulatory risk in communities study (CIRCS)—.* Circulation journal, 2018. **82**(12): p. 3013-3020.
10. de Oliveira Otto, M.C., et al., *Circulating and dietary omega-3 and omega-6 polyunsaturated fatty acids and incidence of CVD in the Multi-Ethnic Study of Atherosclerosis.* Journal of the American Heart Association, 2013. **2**(6): p. e000506.
11. de Goede, J., et al., *N-6 and N-3 fatty acid cholesteryl esters in relation to fatal CHD in a Dutch adult population: a nested case-control study and meta-analysis.* PloS one, 2013. **8**(5): p. e59408.
12. Guallar, E., et al., *A prospective study of plasma fish oil levels and incidence of myocardial infarction in US male physicians.* Journal of the American College of Cardiology, 1995. **25**(2): p. 387-394.
13. Sun, Q., et al., *Blood concentrations of individual long-chain n–3 fatty acids and risk of nonfatal myocardial infarction.* The American journal of clinical nutrition, 2008. **88**(1): p. 216-223.
14. Mozaffarian, D., et al., *Plasma phospholipid long-chain ω-3 fatty acids and total and cause-specific mortality in older adults: a cohort study.* Annals of internal medicine, 2013. **158**(7): p. 515-525.
15. Harris, W.S., et al., *Erythrocyte long-chain omega-3 fatty acid levels are inversely associated*

with mortality and with incident cardiovascular disease: The Framingham Heart Study. Journal of clinical lipidology, 2018. **12**(3): p. 718-727. e6.

16. Harris, W.S., et al., *Blood omega-3 and trans fatty acids in middle-aged acute coronary syndrome patients.* The American journal of cardiology, 2007. **99**(2): p. 154-158.
17. Block, R.C., et al., *EPA and DHA in blood cell membranes from acute coronary syndrome patients and controls.* Atherosclerosis, 2008. **197**(2): p. 821-828.
18. Albert, C.M., et al., *Blood levels of long-chain n–3 fatty acids and the risk of sudden death.* New England Journal of Medicine, 2002. **346**(15): p. 1113-1118.
19. Hamazaki, K., et al., *Plasma levels of n-3 fatty acids and risk of coronary heart disease among Japanese: The Japan Public Health Center-based (JPHC) study.* Atherosclerosis, 2018. **272**: p. 226-232.
20. Liu, Q., et al., *Plasma phospholipid fatty acids and coronary heart disease risk: A matched case-control study within the Women's Health Initiative Observational study.* Nutrients, 2019. **11**(7): p. 1672.

Case-control:

1. Hadj Ahmed, S., et al., *Association of plasma fatty acid alteration with the severity of coronary artery disease lesions in Tunisian patients.* Lipids in Health and Disease, 2017. **16**: p. 1-13.
2. Freije, A., *Fatty acid profile of the erythrocyte membranes of healthy Bahraini citizens in comparison with coronary heart disease patients.* Journal of Oleo Science, 2009. **58**(7): p. 379-388.
3. Yli-Jama, P., et al., *Serum free fatty acid pattern and risk of myocardial infarction: a case-control study.* Journal of internal medicine, 2002. **251**(1): p. 19-28.
4. Kirkeby, K., P. Ingvaldsen, and I. Bjerkedal, *Fatty acid composition of serum lipids in men with myocardial infarction.* Acta Medica Scandinavica, 1972. **192**(1-6): p. 513-519.
5. Lea, E., S. Jones, and D. Hamilton, *The fatty acids of erythrocytes of myocardial infarction patients.* Atherosclerosis, 1982. **41**(2-3): p. 363-369.
6. Lemaitre, R.N., et al., *Endogenous red blood cell membrane fatty acids and sudden cardiac arrest.* Metabolism, 2010. **59**(7): p. 1029-1034.
7. Luostarinena, R., M. Boberg, and T. Saldeen, *Fatty acid composition in total phospholipids of human coronary arteries in sudden cardiac death.* Atherosclerosis, 1993. **99**(2): p. 187-193.
8. Marangoni, F., et al., *Omega-6 and omega-3 polyunsaturated fatty acid levels are reduced in whole blood of Italian patients with a recent myocardial infarction: the AGE-IM study.* Atherosclerosis, 2014. **232**(2): p. 334-338.
9. Oda, E., et al., *A case-control pilot study on n-3 polyunsaturated fatty acid as a negative risk factor for myocardial infarction.* International heart journal, 2005. **46**(4): p. 583-591.
10. Pedersen, J., et al., *Adipose tissue fatty acids and risk of myocardial infarction—a case-control study.* European journal of clinical nutrition, 2000. **54**(8): p. 618-625.
11. Prisco, D., et al., *Increased thromboxane A2 generation and altered membrane fatty acid composition in platelets from patients with active angina pectoris.* Thrombosis research, 1986. **44**(1): p. 101-112.
12. Rhee, Y., et al., *Plasma free fatty acid level patterns according to cardiovascular risk status in postmenopausal women.* Clinica Chimica Acta, 2008. **392**(1-2): p. 11-16.
13. Roberts, T., et al., *Linoleic acid and risk of sudden cardiac death.* Heart, 1993. **70**(6): p. 524-

529.
14. Siscovick, D.S., et al., *Dietary intake and cell membrane levels of long-chain n-3 polyunsaturated fatty acids and the risk of primary cardiac arrest.* Jama, 1995. **274**(17): p. 1363-1367.
15. SKULADOTTIR, G., et al., *Arachidonic Acid Level of Non-esterified Fatty Acids and Phospholipids in Serum and Heart Muscle of Patients with Fatal Myocardial Infarction.* Acta Medica Scandinavica, 1988. **223**(3): p. 233-238.
16. Lopes, C., et al., *Intake and adipose tissue composition of fatty acids and risk of myocardial infarction in a male Portuguese community sample.* Journal of the American Dietetic Association, 2007. **107**(2): p. 276-286.

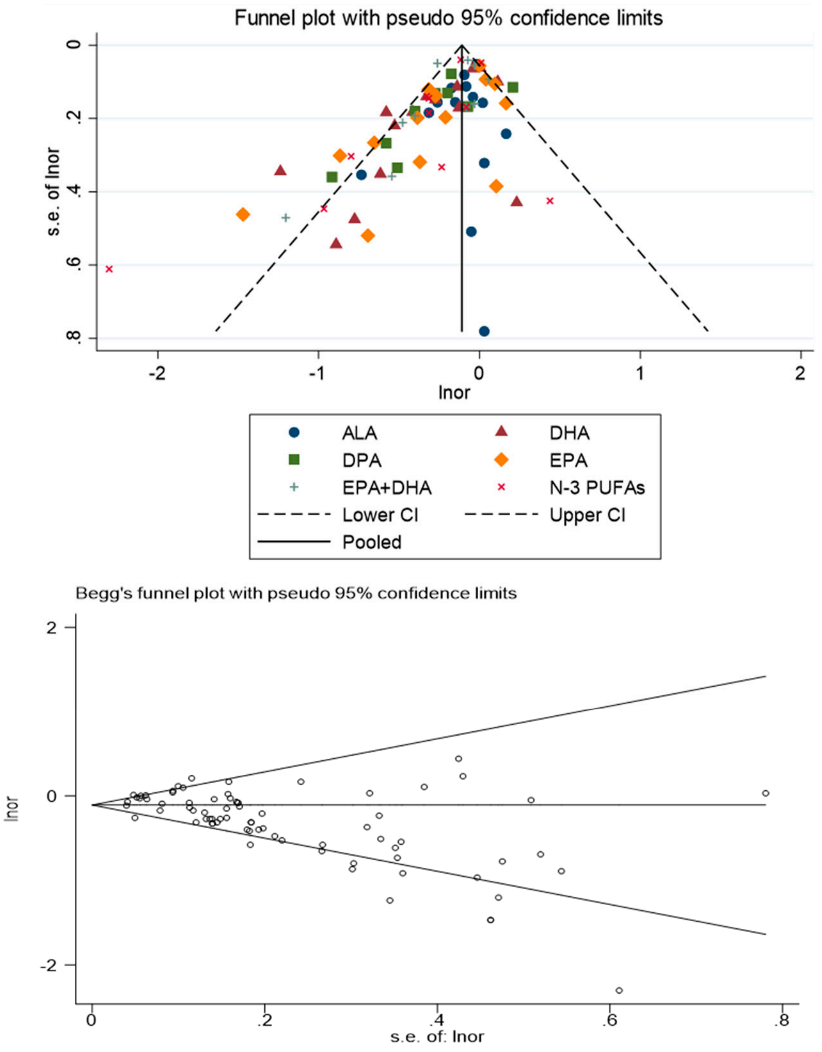

Figure S2 Funnel plot and Begger's test for publication bias of n-3 PUFAs and the risk of coronary heart disease

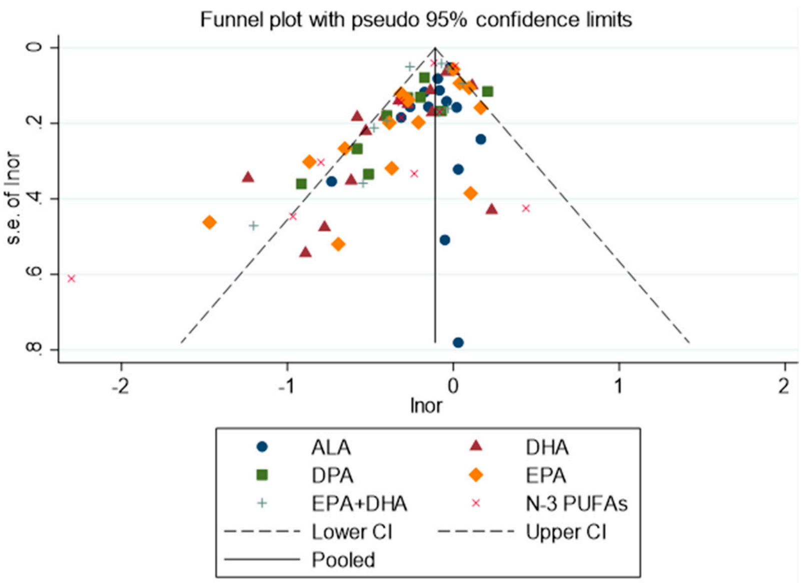

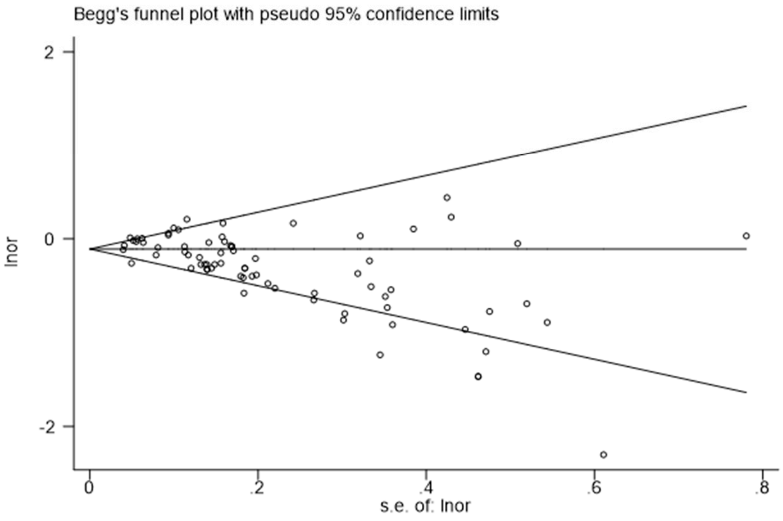

Figure S3 Funnel plot and Begger's test for publication bias of n-3 PUFAs in patients with and without coronary heart disease

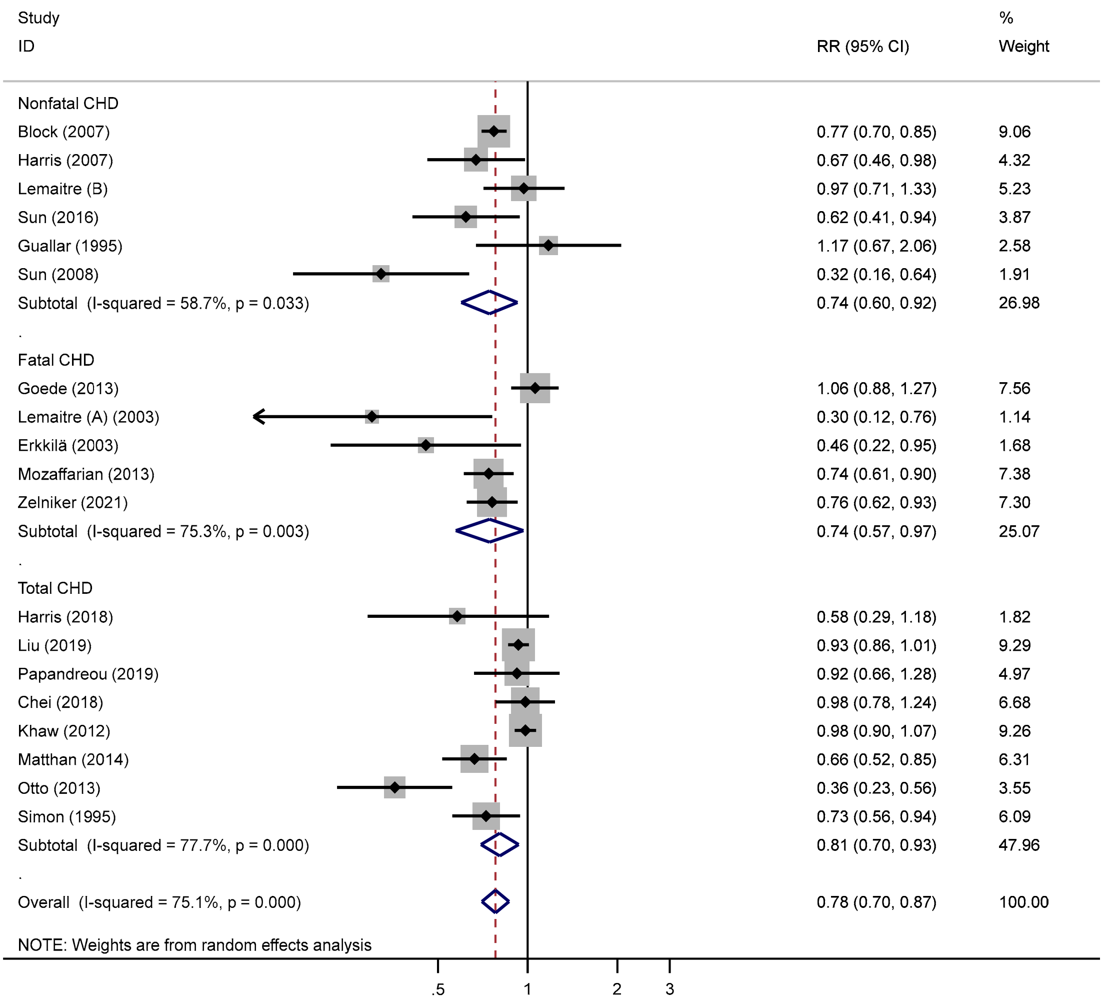

Figure S4 Subgroup for the association between n-3 PUFA with fatal and non-fatal coronary heart disease

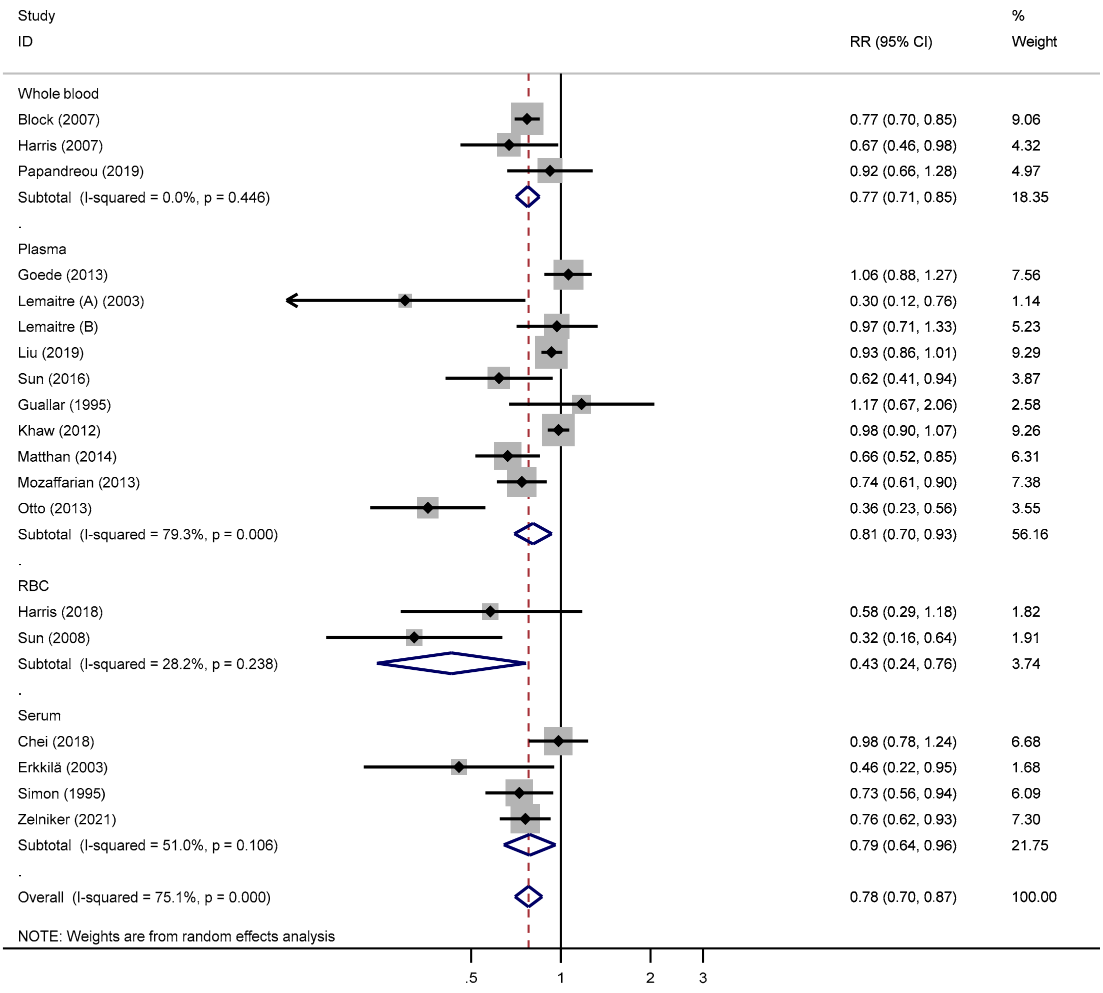

Figure S5 Subgroup for the association between n-3 PUFA compartments and coronary heart disease

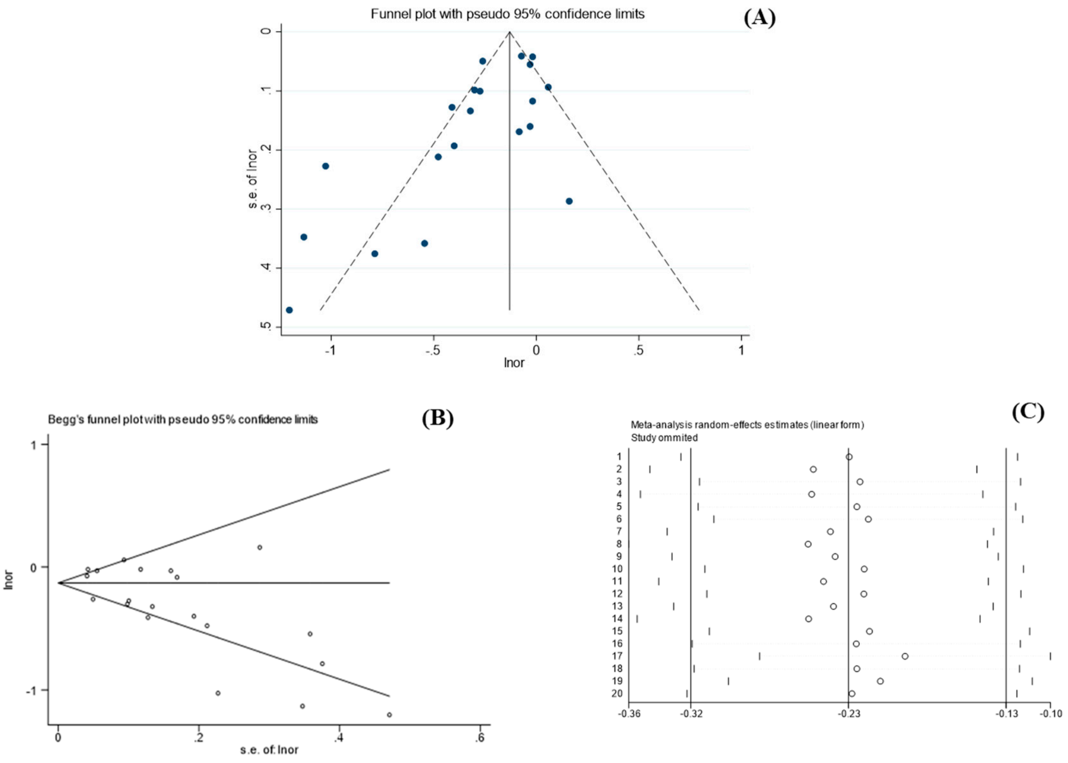

Figure S6 Forest plot (A), begger's test (B), and (C) sensitivity analysis of EPA+DHA and coronary heart disease

Table S3 Meta-regression analysis on CHD and EPA+DHA levels

Moderator variable	p value
**Disease**	
Fatal CHD	0.97
Non-fatal CHD	0.62
Total CHD	0.68
**Biomarker**	
Whole blood	1.00
Plasma	0.61
Erythrocyte	0.91
Serum	0.16

CHD: coronary heart disease; EPA: eicosapentaenoic acid; DHA: docosahexaenoic acid
